# Supplementary material for: Prediction of the Binding to the Nuclear Factor NF-Kappa-B by Constituents from Teucrium polium L. Essential Oil
Source: Curr Issues Mol Biol. 2025 Jan 14;47(1):48. doi: 10.3390/cimb47010048 (PMC11763555; doi:10.3390/cimb47010048)
Supplement: Supplementary file 1 [file cimb-47-00048-s001.zip › cimb-3345630-supplementary.pdf]

Table S1: Smiles codes of *Teucrium polium* molecules drawn in Marvin JS.

Molecules	Smiles
Fenchone	CC1(C2CCC(C2)(C1=O)C)C
3-Carene	CC1=CCC2C(C1)C2(C)C
Limonene oxide, cis-	CC(=C)C1CCC2(C(C1)O2)C
Myrcene	CC(=CCCC(=C)C=C)C
cis-Pinocarveol	CC1(C2CC1C(=C)C(C2)O)C
Germacrene D	CC1=CCCC(=C)C=CC(CC1)C(C)C
Myrtenal	CC1(C2CC=C(C1C2)C=O)C
Bicyclogermacrene	CC1=CCCC(=CC2C(C2(C)C)CC1)C
Myrtenol	CC1(C2CC=C(C1C2)CO)C
Spathulenol	CC1(C2C1C3C(CCC3(C)O)C(=C)CC2)C
(Z)-Nerolidyl acetate	CC(=CCCC(=CCCC(C)(C=C)OC(=O)C)C)C
δ-Cadinene	CC1=CC2C(CCC(=C2CC1)C)C(C)C
β-Ocimene, (E)-	CC(=CCC=C(C)C=C)C
Verbenol	CC1=CC(C2CC1C2(C)C)O
